# Nephroprotective Effect of the Methanolic Extract of *Flourensia cernua* Against Renal Ischemia–Reperfusion Injury Associated with Modulation of Oxidative Stress in Wistar Rats

**DOI:** 10.3390/ijms27198912

**Published:** 2026-10-07

**Authors:** Catalina Leos-Rivas, Carlos Humberto Andrade-Ordaz, Diana Patricia Moreno-Peña, Catalina Rivas-Morales, Liliana Torres-González, Eduardo Sánchez-García, Diana Raquel Rodríguez-Rodríguez, Sandra Loruhama Castillo-Hernández, Linda E. Muñoz-Espinosa, Sergio Arturo Galindo-Rodríguez, Paula Cordero-Pérez

**Affiliations:** 1 Universidad Autonoma de Nuevo Leon, School of Biological Sciences, Department of Analytical Chemistry, San Nicolas de los Garza 66450, Nuevo León, Mexico; catalina.leosrs@uanl.edu.mx (C.L.-R.); carlos.andradeo@uanl.edu.mx (C.H.A.-O.); catalina.rivasmr@uanl.edu.mx (C.R.-M.); eduardo.sanchezgrc@uanl.edu.mx (E.S.-G.); sandra.castillohrn@uanl.edu.mx (S.L.C.-H.); sergio.galindord@uanl.edu.mx (S.A.G.-R.); 2 Universidad Autonoma de Nuevo Leon, University Hospital “Dr. José E. González” Department of Internal Medicine, Liver Unit Monterrey 66460, Nuevo León, Mexico; diana.morenope@uanl.edu.mx (D.P.M.-P.); liliana.torresgzz@uanl.edu.mx (L.T.-G.); diana.rodriguezrgz@uanl.edu.mx (D.R.R.-R.); linda.munozesp@uanl.edu.mx (L.E.M.-E.)

**Keywords:** nephroprotection, renal ischemia–reperfusion, oxidative stress, natural products

## Abstract

Renal ischemia–reperfusion (I/R) injury is a major cause of graft dysfunction. *Flourensia cernua* possesses antioxidant properties that may protect against this injury. This study evaluated its antioxidant capacity and nephroprotective effects in a renal I/R model in Wistar rats. The extract was characterized by phytochemical analysis, total phenolic content, and ABTS, DPPH, and FRAP assays. Hemolytic activity was assessed, and rats were assigned to Sham, Non-Tox, I/R, and I/R + *F. cernua* groups (200 mg/kg/day for 7 days before I/R). Biochemical parameters, renal oxidative stress markers, and *Gpx*, *Rela*, and *Mpo* expression were evaluated. The extract contained several secondary metabolites, showed radical-scavenging and reducing capacity, and had low hemolytic activity. Pretreatment reduced serum creatinine and renal malondialdehyde levels, restored glutathione and superoxide dismutase activity, and downregulated *Gpx* expression compared with I/R. Pretreatment with *F. cernua* partially attenuated I/R-induced renal injury and was associated with reduced lipid peroxidation and preservation of endogenous antioxidant defenses; however, these findings do not establish a causal molecular mechanism.

## 1. Introduction

Chronic kidney disease (CKD) is one of the leading public health problems due to its high morbidity and mortality rates and the substantial economic burden it imposes on healthcare systems. CKD is defined as a reduction in the glomerular filtration rate to less than 60 mL/min/1.73 m^2^, accompanied by structural or functional kidney abnormalities that persist for more than three months [1]. In Mexico, type 2 diabetes mellitus is the leading cause of CKD, followed by arterial hypertension, resulting in a considerable increase in the demand for renal replacement therapies, particularly kidney transplantation [2]. According to the National Transplant Center, kidney transplantation accounts for the largest transplant waiting list in the country, with more than 16,000 registered patients, highlighting the need to develop strategies that improve graft survival and function [3].

Although kidney transplantation is the treatment of choice for end-stage renal disease, it is not without complications. One of the most significant is ischemia–reperfusion (I/R) injury, an unavoidable process during organ procurement, preservation, and implantation. The temporary interruption of blood flow followed by the restoration of circulation triggers a cascade of pathophysiological events, including oxidative stress, inflammation, and cell death, thereby promoting acute kidney injury (AKI) and delayed graft function [4,5].

I/R injury is characterized by excessive production of reactive oxygen species (ROS) and the release of pro-inflammatory cytokines, such as tumor necrosis factor-alpha (TNF-α) and interleukin-6 (IL-6), which contribute to renal functional deterioration [6,7]. Increased oxidative stress promotes lipid peroxidation, protein denaturation, mitochondrial damage, and disruption of multiple metabolic processes, thereby exacerbating renal tissue injury [8,9]. Consequently, modulation of oxidative stress has been investigated as an experimental strategy to attenuate I/R-induced renal damage [10,11].

Several experimental studies have demonstrated that antioxidant compounds, including vitamin C, flavonoids, anthocyanins, and other phenolic compounds, exert protective effects on renal tissue by reducing ROS production, attenuating inflammation, and preserving cellular integrity [12,13,14,15]. Likewise, plant extracts rich in these secondary metabolites have shown nephroprotective activity in different experimental models. For example, an extract of *Myrciaria dubia*, rich in vitamin C and phenolic compounds, reduced serum creatinine levels and preserved renal architecture in rats with gentamicin-induced nephrotoxicity [16]. Previous studies conducted by our group demonstrated that extracts of *Jatropha dioica*, *Sonchus oleraceus*, and *Juglans mollis* exhibit nephroprotective activity, which is primarily attributed to their antioxidant and anti-inflammatory properties [7,17,18].

Although several approaches have been developed to reduce I/R injury, including ischemic preconditioning and the use of pharmacological agents such as simvastatin, aprotinin, and adenosine, their benefits remain limited or are associated with significant adverse effects, including renal failure, cardiovascular events, and anaphylactic reactions [19,20,21,22,23]. Previous studies conducted by our group have demonstrated that pharmacological agents such as misoprostol, as well as metabolites including 2-hydroxyglutarate and succinic acid, attenuate hepatic and renal injury induced by I/R [24,25,26]. Consequently, there is increasing interest in identifying natural compounds with antioxidant and nephroprotective properties that may complement current therapeutic strategies.

Medicinal plants represent an important source of secondary metabolites with relevant biological activities, including antioxidant, anti-inflammatory, and antimicrobial properties [27,28,29]. Among these species, *Flourensia cernua (F. cernua)*, a shrub endemic to northern Mexico and widely distributed in semi-arid regions, has traditionally been used in herbal medicine to treat various gastrointestinal disorders [30]. Phytochemical studies have demonstrated that this species contains high levels of phenolic compounds with potent antioxidant activity and low cytotoxicity [31]. Furthermore, different extracts of *F. cernua* have exhibited antimicrobial activity against clinically important bacteria and fungi, including resistant strains, further supporting its pharmacological potential [32,33,34].

Considering that oxidative stress plays a fundamental role in the pathophysiology of renal I/R injury and that *F. cernua* contains metabolites with recognized antioxidant and anti-inflammatory activities [35], this species is of interest for investigating potential nephroprotective effects in experimental models.

It is important to note that the methanolic extract evaluated in the present renal I/R study was the same extract previously characterized by our research group using liquid chromatography coupled to mass spectrometry (LC-MS). That analysis identified caffeoylquinic and feruloylquinic acid derivatives and flavonoids, including quercetin, kaempferol, genistein, and apigenin glycosides [36]. Thus, the previously established LC-MS profile provides direct chemical characterization of the extract used in the present animal experiment and identifies plausible marker compounds for future mechanistic and quantitative studies.

Therefore, the aim of the present study was to evaluate the antioxidant activity and nephroprotective effect of a methanolic extract of *F. cernua* in an experimental model of renal I/R injury in Wistar rats.

## 2. Results

### 2.1. Extraction Yield

The methanolic extraction process yielded a considerable amount of extract from *F. cernua*. The extraction yield was 32.52%, corresponding to 33.70 g of methanolic extract obtained from 103.60 g of dried plant material.

### 2.2. Phytochemical Profile

The phytochemical screening revealed the presence of several classes of secondary metabolites in the methanolic extract of *F. cernua* (Table 1). The extract contained sterols and triterpenes, coumarins, sesquiterpene lactones, quinones, flavonoids, tannins, and anthrone-type compounds. In contrast, alkaloids and saponins were not detected by the qualitative phytochemical assays employed.

### 2.3. Antioxidant Activity

Table 2 summarizes the chemical radical-scavenging and reducing capacity of the *F. cernua* extract measured by, 2-Diphenyl-1-picrylhydrazyl (DPPH), 2,2′-Azino-bis(3-ethylbenzothiazoline-6-sulfonic acid) diammonium salt (ABTS), and Ferric reducing antioxidant power (FRAP) assays, together with its total phenolic content. The extract showed measurable activity in all three cell-free assays. These findings demonstrate the in vitro chemical antioxidant capacity of the extract, while its antioxidant effects under biological conditions were further evaluated through oxidative stress markers in renal tissue.

### 2.4. Hemolytic Activity

The hemolysis assay revealed a concentration-dependent response of the methanolic extract of *F. cernua* (Table 3). The highest hemolytic activity was observed at 1000 ppm (12.0 ± 3.78%), whereas hemolysis decreased markedly as the extract concentration was reduced. At concentrations of 600 ppm and below, the percentage of hemolysis remained below 1%, reaching a minimum value of 0.09 ± 0.01% at 100 ppm.

### 2.5. Serum Markers of Renal Function

Renal function markers are presented in Figure 1. Comparison between the sham and Non Tox groups revealed no evidence of basal nephrotoxicity associated with the administration of the extract of *F. cernua*, as serum blood urea nitrogen (BUN) and creatinine levels did not differ significantly between the two groups.

Renal I/R induction resulted in AKI, as evidenced by significant increases in serum BUN and creatinine levels compared with the sham group. Treatment with the extract of *F. cernua* partially attenuated I/R-induced renal injury, as indicated by a statistically significant reduction in serum creatinine levels compared with the untreated I/R group. However, serum BUN levels did not differ significantly between the Fc + IR and IR groups.

In addition, serum uric acid concentrations did not differ significantly among the four experimental groups.

### 2.6. Serum Markers of Hepatic Function

Hepatic biochemical parameters are presented in Figure 2. No statistically significant differences were observed among the experimental groups for any of the parameters evaluated.

### 2.7. Oxidative Stress Markers

Renal tissue oxidative stress markers are presented in Figure 3. Renal I/R induction resulted in a significant increase in malondialdehyde (MDA) levels, accompanied by significant decreases in reduced glutathione (GSH) concentrations and superoxide dismutase (SOD) activity compared with the sham group; these direct renal tissue measurements are consistent with increased lipid peroxidation and impaired endogenous antioxidant defenses after I/R.

Pretreatment with the extract of *F. cernua* in the Fc + I/R group significantly reduced renal MDA levels and partially restored GSH concentrations and SOD activity compared with the untreated I/R group. These findings demonstrate an association between extract pretreatment and improved renal redox-related biomarkers under the experimental conditions evaluated.

In addition, comparison of the Non Tox and sham groups revealed significantly lower GSH levels together with significantly higher SOD activity, suggesting that administration of the extract of *F. cernua* modulated the renal redox status in the absence of I/R-induced injury.

### 2.8. Relative Expression of Genes Associated with Renal Injury

Gene expression analysis by qRT-PCR was used as an exploratory assessment of transcriptional responses associated with oxidative stress and inflammation. Pretreatment with the extract of *F. cernua* was associated with a significant reduction in *Gpx* mRNA expression and a nonsignificant trend toward lower *Rela* mRNA expression compared with the I/R group, whereas *Mpo* mRNA expression did not differ significantly among groups (Figure 4).

## 3. Discussion

The present study shows that pretreatment with the methanolic extract of *F. cernua* partially attenuated renal I/R injury in Wistar rats, as evidenced by a reduction in serum creatinine and favorable changes in renal tissue MDA, GSH, and SOD. These findings support an association between the observed nephroprotective response and modulation of redox-related biomarkers. However, because pathway-specific interventions and protein-level analyses were not performed, the present results do not demonstrate that modulation of oxidative stress is the causal mechanism responsible for renal protection.

Phytochemical screening confirmed several classes of secondary metabolites in the *F. cernua* extract reported by Alvides [37]. In addition, the same methanolic extract used in the present animal experiment had previously been characterized by LC-MS by our research group, identifying caffeoylquinic and feruloylquinic acid derivatives and flavonoids including quercetin, kaempferol, genistein, and apigenin glycosides [36]. This previously established profile provides chemical context and improves the reproducibility of the botanical material evaluated here, although quantitative standardization of individual marker compounds was not performed in the present study.

Phenolic compounds have been widely described as effective scavengers of ROS through electron or hydrogen donation [38,39]. In the present study, the extract showed radical scavenging and reducing capacity in the DPPH, ABTS, and FRAP in vitro assays, consistent with its high total phenolic content, which is consistent with previous reports for this plant [40,41]. These findings demonstrate the chemical antioxidant capacity of the extract. In addition, the in vivo redox response was evaluated directly in renal tissue, where pretreatment with *F. cernua* reduced MDA levels and increased GSH levels and SOD activity compared with the I/R group.

Safety evaluation is an essential requirement before considering the pharmacological development of a plant extract. In the present study, the hemolysis assay showed moderate hemolytic activity at the highest concentration evaluated, whereas concentrations equal to or below 600 ppm exhibited less than 1% hemolysis, indicating low cytotoxicity, which is consistent with previous reports for this plant [42,43]. In addition, the Non Tox group showed no significant changes in serum BUN, creatinine, or the evaluated hepatic biochemical parameters compared with Sham. These findings support tolerability under the specific experimental conditions and duration tested, but they should not be interpreted as a comprehensive toxicological assessment.

In the present study, animals subjected to I/R exhibited a significant increase in creatinine and BUN levels compared with the sham group, supporting the establishment of acute renal dysfunction. Pretreatment with the extract of *F. cernua* partially attenuated this functional alteration by significantly reducing serum creatinine, whereas BUN remained unchanged relative to I/R group. These findings are consistent with those previously reported by our group, demonstrating that the administration of plant extracts with antioxidant activity significantly reduces serum creatinine; however, in the case of BUN, certain extracts may either significantly decrease or have no effect on this marker in experimental models of renal I/R injury [7,18]. The functional evidence supports a partial nephroprotective effect rather than complete preservation of renal function. More sensitive injury biomarkers such as Neutrophil Gelatinase-Associated Lipocalin (NGAL) or Kidney Injury Molecule-1(KIM-1) [44] and urinary parameters were not measured and should be incorporated in future studies.

Given that AKI can induce distant organ dysfunction, we next examined whether renal I/R led to secondary hepatic impairment. Renal I/R induction did not alter the serum levels of alanine aminotransferase (ALT), aspartate aminotransferase (AST), or lactate dehydrogenase (LDH) compared with the sham group, suggesting the absence of secondary hepatocellular injury or hepatic necrosis following renal I/R. Likewise, markers of hepatic synthetic function, including total protein and albumin concentrations, as well as alkaline phosphatase (ALP) activity, remained unchanged across all experimental groups, indicating that neither the I/R procedure nor treatment with the extract of *F. cernua* adversely affected liver function, which is consistent with previous reports [7,17,18].

The protective effect on renal function was accompanied by significant changes in oxidative stress markers. The significant increase in MDA levels observed in the I/R group suggests an increase in lipid peroxidation during reperfusion, a process considered one of the main mechanisms responsible for tissue damage. Likewise, the reduction in SOD and GSH activity reflects the depletion of cellular antioxidant defenses. Pretreatment with *F. cernua* significantly reduced MDA concentrations and restored SOD and GSH antioxidant activity, suggesting that the extract limits the propagation of oxidative damage and promotes redox homeostasis. These findings are consistent with those reported by other authors, who observed that administration of *Ginkgo biloba, Juglans mollis*, and *Sonchus oleraceus* extracts in a renal I/R model reduced MDA levels and increased antioxidant enzyme activity [17,18,45].

The evaluation of gene expression enabled the exploration of molecular mechanisms underlying the response to treatment. In line with the changes observed in biochemical and oxidative stress markers. During reperfusion, ROS can activate redox-sensitive transcriptional responses involving *Nrf2* and *NF-κB* and can alter antioxidant genes such as members of the *GPx* family [46,47]. In the present study, *Gpx* mRNA expression was significantly reduced by *F. cernua* pretreatment relative to I/R, but mRNA abundance does not necessarily reflect GPx protein abundance or enzymatic activity. Nrf2/HO-1 signaling was not directly evaluated.

On the other hand, the *Rela* expression showed a decreasing trend in animals pretreated with *F. cernua* extract; the differences did not reach statistical significance. Likewise, *Mpo* mRNA did not differ significantly among groups. Accordingly, these findings do not provide sufficient evidence to conclude that the extract exerted an anti-inflammatory effect through NF-κB or neutrophil MPO pathways. Future studies should include protein-level or activity measurements of NF-κB/p65 and MPO, together with inflammatory mediators such as TNF-α, Interleukin-1β, IL-6, and Monocyte Chemoattractant Protein-1, to determine whether inflammatory signaling contributes to the observed renal protection.

In this context, our previous LC-MS profile obtained from the same extract, which was used in this study, identified caffeoylquinic and feruloylquinic acid derivatives and flavonoids such as quercetin, kaempferol, genistein, and apigenin glycosides [36]. These compounds are plausible contributors to the observed biological response based on their reported redox and inflammatory activities [48]; however, the present study did not isolate individual constituents or establish compound-specific causality. Future fractionation, quantitative marker standardization, and activity-guided studies are required to identify the constituents responsible for the nephroprotective effect.

One of the main strengths of the present study is the comprehensive evaluation of the extract, which included its phytochemical characterization, antioxidant capacity, safety profile, renal function assessment, oxidative stress markers, and gene expression analysis in an experimental model of renal I/R injury. However, several limitations should be considered. Only one extract dose (200 mg/kg/day) and one IR time were evaluated, preventing assessment of dose–response relationships and temporal effects. The renal functional assessment was based on serum creatinine, BUN, and uric acid; more sensitive biomarkers such as NGAL or KIM-1 and urinary parameters were not included. Histopathological evaluation was not performed. Furthermore, the molecular assessment was limited to mRNA expression of *Gpx*, *Rela*, and *Mpo*, without protein quantification, immunohistochemistry, cytokine measurements, or direct evaluation of Nrf2/HO-1 and NF-κB pathway activation. These limitations preclude definitive mechanistic conclusions and should be addressed in future studies.

## 4. Materials and Methods

The plant material of *F. cernua* was purchased from the commercial supplier Pacalli Company, Guadalupe, Nuevo León, México. The hydroalcoholic extract of *F. cernua* was kindly provided by Dr. Catalina Leos (School of Biological Sciences, Universidad Autónoma de Nuevo Leon). The methanolic extract used throughout the present study was the same extract previously characterized by LC-MS [36]. The aerial parts of the plant were ground to obtain a homogeneous particle size. The powdered material was subsequently subjected to static maceration extraction using methanol as the extraction solvent for seven days at room temperature under light-protected conditions. After extraction, the mixture was filtered, and the solvent was removed under reduced pressure using a rotary evaporator to obtain the dried methanolic extract, which was stored at 4 °C until further use in the different assays.

### 4.1. Qualitative Phytochemical Profile

The phytochemical profile of the methanolic extract of *F. cernua* was determined using conventional colorimetric assays for the qualitative identification of the major groups of secondary metabolites.

Alkaloids were detected using Dragendorff’s reagent; flavonoids were identified by the Shinoda test; sesquiterpene lactones by the Baljet test; carbohydrates by the Molisch reaction; sterols and triterpenes by the Liebermann–Burchard and Salkowski tests; phenolic compounds (tannins) by the ferric chloride reaction; and unsaturated compounds by the potassium permanganate test. The presence of each phytochemical group was established based on the characteristic color changes or precipitate formation described for each assay [49,50].

### 4.2. Determination of Antioxidant Activity

#### 4.2.1. DPPH Radical Scavenging Assay

The free radical scavenging activity of the methanolic extract of *F. cernua* was evaluated using the DPPH assay (Sigma Aldrich, St. Louis, MO, USA) and the FRAP assay (Sigma Aldrich, St. Louis, MO, USA) both determined spectrophotometrically [51,52].

For the DPPH assay, a 280 μM DPPH solution prepared in absolute methanol, quercetin (1 mM) as the positive control, and different concentrations of the methanolic extract (6.25–1000 μg/mL) were used. Briefly, 100 μL of each extract concentration was mixed with 100 μL of the DPPH solution in a 96-well microplate. The reaction mixture was incubated in the dark at room temperature for 15 min, after which absorbance was measured at 540 nm using a Multiskan GO Microdrop spectrophotometer (Thermo Fisher Scientific, Carlsbad, CA, USA).

Both the calibration curves and sample analyses were performed in triplicate. The obtained data were plotted to generate the linear regression equation (y = mx + b), which was used to calculate the amount of Trolox (Sigma Aldrich, St. Louis, MO, USA) equivalents (nmol) present in each sample aliquot. The Trolox Equivalent Antioxidant Capacity was expressed as μmol Trolox equivalents per gram of dry weight (μmol TE/g DW).

#### 4.2.2. Ferric Reducing Antioxidant Power Assay

The antioxidant capacity of the samples was determined using the FRAP assay as previously described by Viveros-Valdez [51]. For the assay, 300 μL of the FRAP working reagent was added to each well of a 96-well microplate, followed by 10 μL of sample and 30 μL of distilled water (final dilution 1:34). The initial absorbance was recorded at 593 nm, and the reaction mixture was incubated for 1 h at room temperature. The final absorbance was then measured at the same wavelength.

A stock solution of Trolox was prepared by dissolving 0.01 g of Trolox in methanol/distilled water (1:1, *v*/*v*), and serial dilutions were prepared to obtain concentrations ranging from 79 to 399 μM.

The antioxidant activity by DPPH and ABTS was expressed as half-maximal effective concentration 50; DPPH was expressed as μmol of Trolox equivalent per gram of extract (μmol TE/g); FRAP was expressed as micromoles of Fe^2+^ equivalents per gram of dry extract; and flavonoids were expressed in milligrams of gallic acid equivalents (GAE) per gram of the hydroalcoholic extract of *F. cernua*.

#### 4.2.3. Determination of Total Antioxidant Capacity by ABTS

For the ABTS antioxidant capacity, the procedure detailed by Granados-Guzmán [53] was performed, using quercetin (Sigma Aldrich, St. Louis, MO, USA) as a positive control. The ABTS radical cation (ABTS^+^) was generated by reacting a 7 mM ABTS solution (Sigma Aldrich, St. Louis, MO, USA) with 2.45 mM potassium persulfate(Sigma Aldrich, St. Louis, MO, USA, and the mixture was incubated for 16–18 h in the dark. After this period, the solution was diluted with 70% ethanol (Sigma Aldrich, St. Louis, MO, USA) to reach an absorbance of 0.7 ± 0.2. Subsequently, 60 μL of the extract or quercetin at different concentrations (yielding 20–80% inhibition of the blank absorbance) was mixed with 140 μL of the ABTS^+^ working solution, incubated for 6 min in the dark, and the absorbance was measured at 750 nm using a spectrophotometer. For the blank control, 70% ethanol was used instead of the sample. The percentage of inhibition was calculated according to the equation, and the half-maximal effective concentration (EC_50_), defined as the concentration required to produce a 50% reduction in the ABTS^+^ radical, was interpolated for both the extract and quercetin.

$$\%\;\mathrm{of reduction}\;=\frac{(\mathrm{Abs control}-\mathrm{Abs sample})\times 100}{\mathrm{Abs control}}$$

#### 4.2.4. Determination of Total Phenolic Content

The total phenolic content of the methanolic extract was determined using the Folin–Ciocalteu reagent according to the method described by Viveros-Valdez [51]. Briefly, 50 μL of the sample was added to a microplate, followed by 100 μL of 1 N Folin–Ciocalteu reagent, 100 μL of 10% sodium carbonate (Sigma Aldrich, St. Louis, MO, USA), and 400 μL of distilled water. The reaction mixture was incubated at 37 °C for 30 min, and absorbance was measured at 765 nm using a spectrophotometer. Gallic acid (Sigma Aldrich, St. Louis, MO, USA) was used as the calibration standard. Total phenolic content was expressed as milligrams of GAE per gram of dry extract. All measurements were performed in triplicate in three independent experiments.

### 4.3. Evaluation of Hemolytic Activity

The biological safety of the methanolic extract of *F. cernua* was evaluated through an in vitro hemolysis assay using human O Rh-positive erythrocytes obtained in accordance with the Mexican Official Standard NOM-253-SSA1-2012. This assay is widely employed to assess the toxicity of plant extracts intended for biological activity studies in murine models, as it measures the extent of erythrocyte membrane disruption, a critical indicator of cytotoxic potential [54,55]. The assay was performed following the methodology described by Elizondo-Luevano [56]. Whole blood was centrifuged at 3000 rpm for 15 min at room using a centrifuge sorvall st8 (Thermo Fisher Scientific, Carlsbad, CA, USA) temperature to obtain the erythrocyte pellet, which was washed four times with phosphate-buffered saline (Sigma Aldrich, St. Louis, MO, USA), (1 × PBS, pH 7.4). The methanolic extract was dissolved in PBS to obtain final concentrations of 100, 200, 400, 600, 800, and 1000 μg/mL (*w*/*v*), and erythrocytes were incubated with each concentration for 30 min at 37 °C under light-protected conditions. After incubation, samples were centrifuged at 13,000 rpm for 5 min at 4 °C, and the supernatants were collected for spectrophotometric determination of hemoglobin release at 540 nm using a spectrophotometer. All experiments were performed in triplicate using 96-well microplates. The percentage of hemolysis was calculated by comparing the absorbance of treated samples with that of the positive control (complete erythrocyte lysis) according to the equation Hemolysis (%) = (Abs_540_ Treatment/Abs_540_ Positive Control) × 100, while phosphate-buffered saline served as the negative control.

### 4.4. Evaluation of Nephroprotective Activity

#### 4.4.1. Experimental Design

The nephroprotective activity of the methanolic extract of *F. cernua* was evaluated using an experimental model of renal I/R injury in Wistar rats, which has been widely used to evaluate said activity [17,25].

1. Sham: Animals received 2% Tween 20 (Sigma Aldrich, St. Louis, MO, USA), (1 mL/day) by intragastric administration for seven consecutive days and underwent laparotomy without renal I/R induction.
2. Non Tox: Animals received the extract of F. cernua (200 mg/kg/day in 2% Tween 20 intragastrically) for seven consecutive days and underwent laparotomy without I/R induction.
3. I/R: Animals received 2% Tween 20 for seven consecutive days and were subsequently subjected to bilateral renal ischemia for 60 min followed by 15 h of reperfusion.
4. Fc + I/R: Animals were pretreated with the extract of F. cernua (200 mg/kg/day in 2% Tween 20 intragastrically) for seven consecutive days before bilateral renal ischemia followed by reperfusion.

#### 4.4.2. Experimental Animals

Twenty-four male Wistar rats weighing between 220 and 300 g were used in this study. Animals were housed under standard laboratory conditions (22 ± 2 °C, 50–60% relative humidity, and a 12 h light/dark cycle) with free access to water and commercial chow. Rats were acquired from Nutrimix de México, S.A. de C.V. (Mexico City, México).

Only clinically healthy animals with body weights within the established range were included. Rats older than six months of age were excluded. In addition, animals that died during the experimental procedures or presented infectious diseases or evident renal morphological abnormalities were excluded from the study.

#### 4.4.3. Renal I/R Injury Model

Animals were anesthetized by intraperitoneal administration of ketamine (100 mg/kg, Anesket, PiSA Agropecuaria, Mexico) and xylazine (10 mg/kg, Sedaject, Vedilab, Mexico). A midline laparotomy was then performed under aseptic conditions.

In the sham and Non Tox groups, both kidneys were exposed without vascular occlusion. The abdominal cavity remained open for 60 min to simulate the surgical procedure, after which the abdominal wall was closed in layers using continuous 5-0 polypropylene sutures.

In the I/R and Fc + I/R groups, both renal pedicles were carefully dissected and occluded with atraumatic vascular clamps for 60 min to induce bilateral renal ischemia. After the ischemic period, the clamps were removed to allow reperfusion for 15 h. Subsequently, the abdominal wall was closed in anatomical layers using continuous 5-0 polypropylene sutures.

During the postoperative period, animals were continuously monitored, provided with external heat support, allowed free access to food and water, and received tramadol for postoperative analgesia.

#### 4.4.4. Collection of Biological Samples

Following the reperfusion period, the animals were re-anesthetized with ketamine and xylazine for biological sample collection by exsanguination via abdominal aorta puncture.

Blood samples were allowed to clot at room temperature and subsequently centrifuged at 3500 rpm for 15 min to obtain serum.

Both kidneys were excised, rinsed with cold saline solution, and longitudinally sectioned. A portion of each kidney was stored at −80 °C for subsequent determination of oxidative stress biomarkers and gene expression analysis.

### 4.5. Determination of Biochemical Markers

Serum samples were analyzed using an automated biochemical analyzer (ILAB Aries^®^, Instrumentation Laboratory, Rome, Italy) to determine serum creatinine, BUN, uric acid, ALT, AST, LDH, ALP, total protein, and albumin levels, using the manufacturer’s recommended reagents.

### 4.6. Determination of Oxidative Stress Biomarkers

Renal tissue samples were homogenized under cold conditions in the presence of specific lysis buffers for the evaluation of oxidative stress biomarkers: radioimmunoprecipitation assay buffer for MDA determination, 4-(2-hydroxyethyl)-1-piperazineethanesulfonic acid buffer for SOD activity, and potassium phosphate buffer for reduced GSH quantification. All procedures were performed on ice to prevent oxidative degradation.

MDA levels, as an indicator of lipid peroxidation, were determined using the thiobarbituric acid reactive substances assay (Cayman Chemical Company, Ann Arbor, MI, USA), based on the formation of the MDA–thiobarbituric acid complex. After centrifugation, the absorbance of the reaction product was measured at 535 nm using a using a spectrophotometer.

SOD activity was evaluated using a colorimetric assay based on the inhibition of tetrazolium reduction by the xanthine/xanthine oxidase system (Cayman Chemical Company, Ann Arbor, MI, USA), with absorbance measured at 450 nm using a spectrophotometer. GSH levels were quantified through the reduction of 5,5′-dithiobis(2-nitrobenzoic acid) to 5-thio-2-nitrobenzoic acid using a commercial GSH assay kit (Sigma-Aldrich, St. Louis, MO, USA), and absorbance was recorded at 412 nm using a spectrophotometer.

The levels of oxidative stress biomarkers were normalized to the total protein content of tissue homogenates, determined using the Bradford assay [57]. Results are expressed as nmol/mg protein for MDA, U/mg protein for SOD activity, and nmol/mg protein for GSH levels.

### 4.7. Gene Expression Analysis by RT-qPCR

Total RNA was extracted from 100 mg of frozen kidney tissue using TRIzol reagent (Invitrogen, Thermo Fisher Scientific, Carlsbad, CA, USA) according to the manufacturer’s instructions. RNA concentration was determined spectrophotometrically using a using a spectrophotometer.

Gene expression analysis was performed using a StepOne™ Real-Time PCR System (Thermo Fisher Scientific) and a One-Step qRT-PCR Kit (Promega Corporation, Madison, WI, USA). Each reaction contained 100 ng of total RNA and primers at final concentrations ranging from 50 to 300 nM, following the manufacturer’s protocol. The primer sequences used were as follows: *Rela*: forward 5′-CCTCATCTTTCCCTCAGAGCC-3′; reverse 5′-CGCACTTGTAACGGAAACGC-3′; *Gpx*: forward 5′-CATTGAGAATGTCGCGTCCC-3′; reverse 5′-TTGCCATTCTCCTGATGTCCG-3′; *Mpo*: forward 5′-ATGTCACAGGGGACATGCG-3′; reverse 5′-TCTGTGGCCAGACGGTTATG-3′; *Actb* (endogenous reference gene): forward 5′-CCCTGGCTCCTAGCACCAT-3′; reverse 5′-GATAGAGCCACCAATCCACACA-3′.

Thermal cycling conditions consisted of one reverse transcription cycle at 48 °C for 15 min, followed by reverse transcriptase inactivation and DNA polymerase activation at 95 °C for 10 min. Amplification was performed for 40 cycles of denaturation at 95 °C for 10 s and annealing and extension at 60 °C for 30 s. Relative gene expression levels were calculated using the comparative 2^−ΔΔCt^ method.

### 4.8. Statistical Analysis

For the in vivo study, the experimental unit and biological replicate was the individual animal (n = 6 animals per group). Measurements performed repeatedly on the same sample or well were considered technical replicates and were not treated as independent biological observations. For the in vitro assays, replicate measurements were used to estimate assay precision as described in the corresponding methods. A Shapiro–Wilk test was performed to assess data normality. Data meeting the parametric assumption are expressed as mean ± standard deviation (SD). Statistical differences among groups were analyzed by one-way analysis of variance (ANOVA), followed by Tukey’s multiple-comparisons post hoc test. Statistical analyses were performed using GraphPad Prism version 8.0 (GraphPad Software, San Diego, CA, USA). Differences were considered statistically significant at *p* < 0.05.

### 4.9. Ethical Considerations

All animal experimental procedures were conducted in accordance with internationally accepted guidelines for the care and use of laboratory animals and with current Mexican regulations governing biomedical research (NOM-062-ZOO-1999). The experimental protocols were approved by the Research Ethics Committee and Research Committee UANL (CONBIOÉTICA-19-CEI-001–20160404) and the Institutional Animal Care and Use Committee of the corresponding institution (approval protocol number QA24-00001).

Human blood samples used for the hemolysis assay were obtained in compliance with the Mexican Official Standard NOM-253-SSA1-2012 and with prior approval from the corresponding Institutional Ethics Committee.

## 5. Conclusions

In conclusion, pretreatment with the methanolic extract of *F. cernua* partially attenuated renal I/R injury under the experimental conditions evaluated, as shown by reduced serum creatinine and favorable changes in renal tissue MDA, GSH, and SOD. These findings support an association between nephroprotection and preservation of renal redox homeostasis but do not establish a causal molecular mechanism. Because the extract was administered before ischemia, the results specifically support a preventive/preconditioning effect rather than treatment of established I/R injury. The same extract was previously characterized by LC-MS, identifying phenolic acid derivatives and flavonoids that represent plausible candidates for future activity-guided studies. Additional dose–response experiments, sensitive renal injury biomarkers, urinary and histopathological assessments, inflammatory mediators, and protein-level evaluation of pathways such as Nrf2/HO-1 and NF-κB are required to define the mechanism and translational relevance.

## Figures and Tables

**Figure 1:**
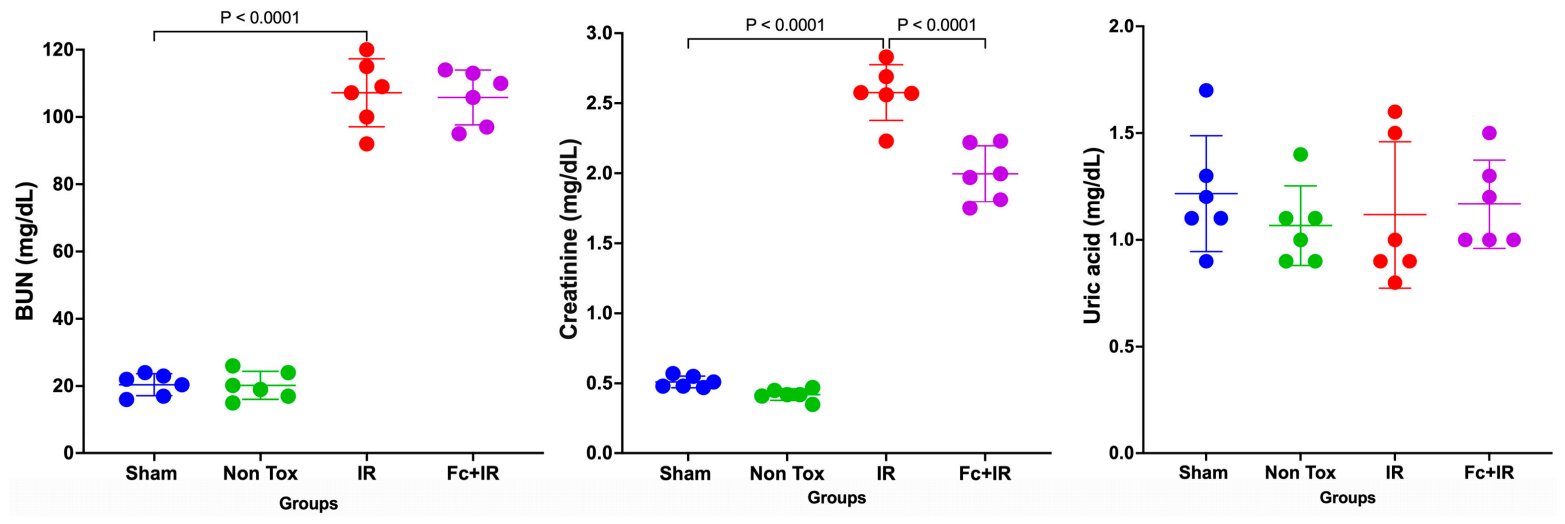
Renal function parameters in the experimental groups. Sham: untreated animals without renal injury; Non Tox: animals treated with the extract of *F. cernua* without renal injury; I/R: ischemia–reperfusion; Fc + I/R: animals treated with the extract of *F. cernua* prior to renal I/R. Data are presented as the mean ± SD. Statistical differences among groups were analyzed using ANOVA followed by Tukey’s multiple-comparison post hoc test.

**Figure 2:**
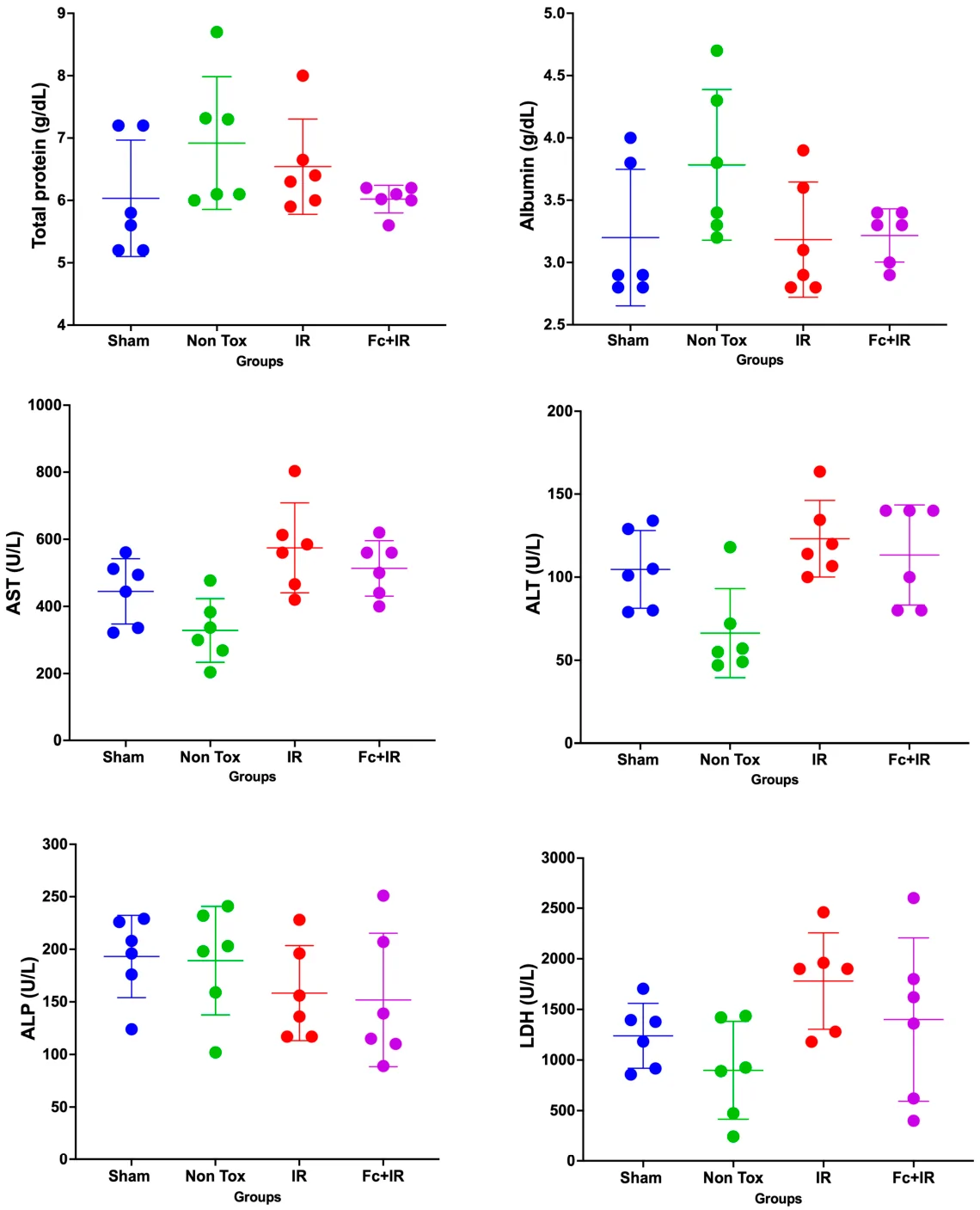
Hepatic biochemical parameters in the experimental groups. Sham: untreated animals without renal injury; Non Tox: animals treated with the extract of *F. cernua* without renal injury; I/R: ischemia–reperfusion; Fc + I/R: animals treated with the extract of *F. cernua* prior to renal I/R. AST: *aspartate aminotransferase*; ALT: *alanine aminotransferase*; ALP: *alkaline phosphatase*; LDH: *lactate dehydrogenase*. Data are expressed as the mean ± SD. Statistical differences among groups were analyzed using ANOVA followed by Tukey’s multiple-comparison post hoc test.

**Figure 3:**
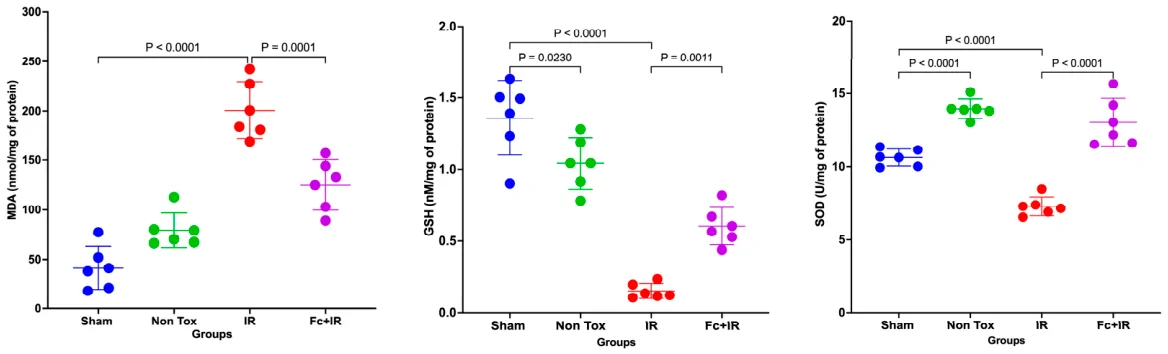
Renal tissue oxidative stress parameters in the experimental groups. Sham: untreated animals without renal injury; Non Tox: animals treated with the extract of *F. cernua* without renal injury; I/R: ischemia–reperfusion; Fc + I/R: animals treated with the extract of *F. cernua* prior to renal I/R. Data are expressed as the mean ± SD. Statistical differences among groups were analyzed using ANOVA followed by Tukey’s multiple-comparison post hoc test.

**Figure 4:**
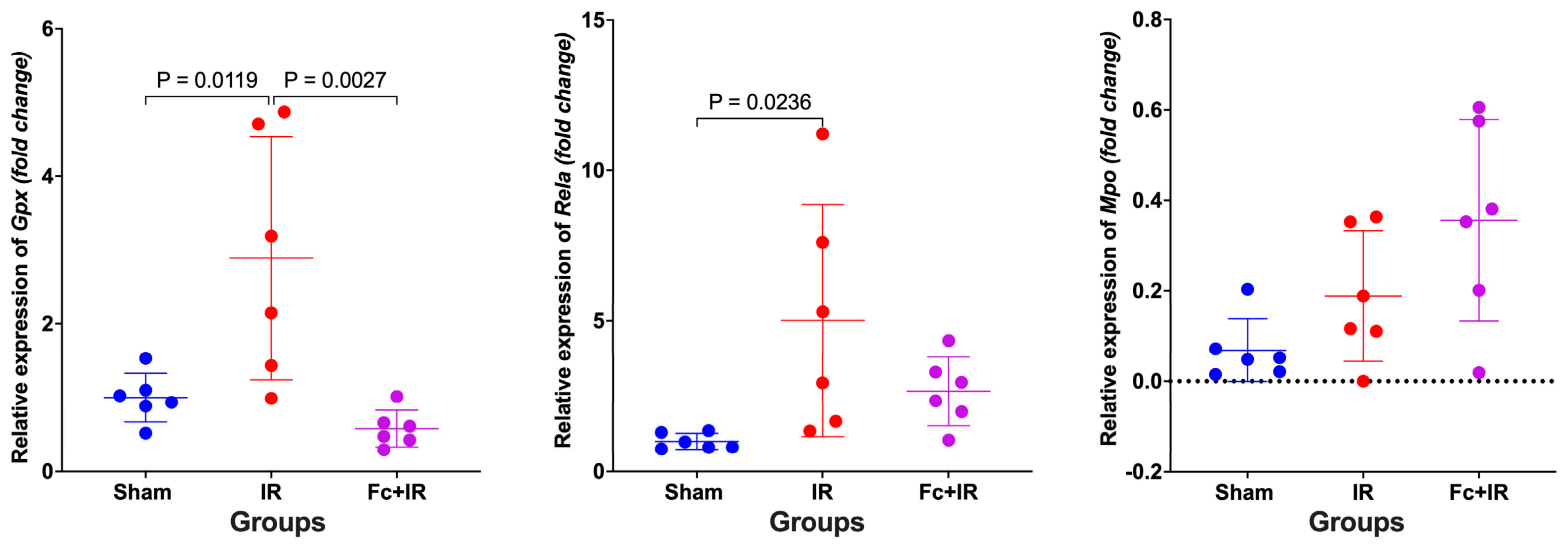
Relative expression of genes associated with renal injury. Sham: untreated animals without renal injury; Non Tox: animals treated with the extract of *F. cernua* without renal injury; I/R: ischemia–reperfusion; Fc + I/R: animals treated with the extract of *F. cernua* prior to renal I/R; *Gpx*: glutathione peroxidase; *Mpo*: myeloperoxidase; *Rela*: nuclear factor kappa B (NF-κB; in rats, encoded by the *Rela* gene). Data are expressed as the mean ± SD. Statistical differences among groups were analyzed using ANOVA followed by Tukey’s multiple-comparison post hoc test.

**Table 1:** Qualitative phytochemical profile of the methanolic extract of *F. cernua*.

Phytochemical Test	Metabolite Detected	*F. cernua*
Liebermann–Burchard	Sterols and triterpenes	+
Coumarin test	Coumarins	+
Baljet	Sesquiterpene lactones	+
Quinone test	Quinones	+
Foam test	Saponins	−
Shinoda	Flavonoids	+
Ferric chloride test	Tannins	+
Anthrone test	Anthrone-type compounds	+
Dragendorff	Alkaloids	−

Note: (+) Test positive, (−) Test negative.

**Table 2:** Antioxidant activity by DPPH, ABTS, FRAP and flavonoid content.

	DPPH		ABTS	FRAP	Flavonoids
Sample	CE_50_ (μg/mL)	μmol TE/g	CE_50_ (μg/mL)	μmol FeE/g	mg GAE/g
Quercetin	3.68 ± 0.16	4.7 ± 0.56	205.21 ± 0.42	Not determined	Not determined
*F. cernua*	337.46 ± 1.51	125.03 ± 2.16	329.89 ± 0.47	292.59 ± 1.54	4708.60 ± 0.24

CE50: half-maximal effective concentration 50; TE: Trolox equivalent; GAE: gallic acid equivalent; FeE: Fe^2+^ iron equivalents.

**Table 3:** Hemolytic activity of the methanolic extract of *F. cernua*.

Concentration (ppm)	Hemolysis (%) *F. cernua*
1000	12.00 ± 3.78
800	2.64 ± 1.45
600	0.37 ± 0.13
400	0.73 ± 0.07
200	1.29 ± 0.02
100	0.09 ± 0.01

## Data Availability

The data that support the findings of this study are available from the corresponding author upon reasonable request.

## Abbreviations

The following abbreviations are used in this manuscript:

ABTS	2,2′-Azino-bis(3-ethylbenzothiazoline-6-sulfonic acid) diammonium salt
ALP	Alkaline phosphatase
ALT	Alanine aminotransferase
AST	Aspartate aminotransferase
BUN	Blood urea nitrogen
CKD	Chronic kidney disease
DPPH	2,2-Diphenyl-1-picrylhydrazyl
FRAP	Ferric reducing antioxidant power
GAE	Milligrams of gallic acid equivalents
GSH	Glutathione
I/R	Ischemia–reperfusion
LDH	Lactate dehydrogenase
MDA	Malondialdehyde
ROS	Reactive oxygen species
SOD	Superoxide dismutase
